# Situation-Based Survey of Avian Influenza Viruses in Possible “Bridge” Species of Wild and Domestic Birds in Nigeria

**DOI:** 10.1155/2012/567601

**Published:** 2012-09-02

**Authors:** Vakuru Columba Teru, Shiiwua A. Manu, Gashash I. Ahmed, Kabir Junaidu, Scott Newman, Joseph Nyager, Vivian N. Iwar, Gideon M. Mshelbwala, T. Joannis, Junaidu A. Maina, Paul T. Apeverga

**Affiliations:** ^1^Epidemiology Unit, Department of Livestock, Federal Ministry of Agriculture, Area 11, P.M.B 135, Garki, Abuja, Nigeria; ^2^A.P. Leventis Ornithological Research Institute (APLORI), University of Jos Biological Conservatory, P.O. Box 13403, Laminga, Plateau State, Jos, Nigeria; ^3^Department of Veterinary Public Health, Faculty of Veterinary Medicine, Ahmadu Bello University (ABU), Zaria, Nigeria; ^4^EMPRES Animal Health-Wildlife Health & Ecology Unit, FAO, Rome, Italy; ^5^Department of Agriculture and Rural Development, ECOWAS Commission, 60 Yakubu Gown Crescent, Asokoro District, P.M.B 401, Abuja, Nigeria; ^6^National Veterinary Research Institute (NVRI), Plateau State, Vom, Nigeria

## Abstract

The highly pathogenic avian influenza (H5N1 subtype) recurred in Nigeria after 9 months period of no reported case. A critical look at possible sources of the re-occurrence was desirable. The objective of this study was to determine whether avian influenza viruses were present at reasonably detectable levels (0.5%) in possible “bridge” species of wild and domestic birds. The study was conducted in 8 Nigerian states. A total of 403 birds from 40 species were sampled. Virus isolation was done in embryonated chicken eggs according to standard protocols. The test results were all negative for avian influenza viruses. The overall confidence interval (CI) calculated in R using the exact binomial confidence interval function was 0–0.007406. Tawny Eagle (*Aquila rapax*) was the lowest sampled 0.3% (1/403) and Red-billed Firefinch (*Lagonosticta senegala*) the highest 11.7% (47/403). The limitations of the sample size and possibly designing effects on the study, as to make concrete conclusions were acknowledged. Species of wild birds, so identified in the study could be useful in future surveys. Furthermore, multidisciplinary and community oriented approach, blending targeted and passive surveillances was suggested. This approach was envisaged to bring about wider coverage of “bridge” species and clearer insight of their possible roles in avian influenza re-occurrences and spread in Nigeria.

## 1. Introduction 

Avian influenza (AI) is a highly contagious disease primarily of birds, and caused by influenza A viruses. Influenza A viruses in poultry can be grouped into 2. The exceptionally virulent viruses cause highly pathogenic avian influenza (HPAI), with mortality in affected flock as high as 100%. This group belongs to subtypes H5 and H7, but it is worth noting that not all H5 and H7 viruses' infection lead to HPAI. All other subtypes cause a milder, primarily respiratory, disease, that is, low pathogenic avian influenza (LPAI) unless exacerbated by secondary infections [1]. Avian influenza is one of the greatest concerns for public health that has emerged from the animal reservoir [2]. The spread of HPAI (H5N1) to countries in which hygienic standards are deficient increases the virus's pandemic potential and raises concerns about food security particularly in rural villages [2]. Aquatic birds are the sources of avian influenza viruses [3, 4]. Aquatic birds like ducks, geese, swans (Anseriformes), and gulls, terns (Charadriiforms) are thought to be natural reservoirs for avian influenza viruses and are capable of shading the viruses asymptomatically [5, 6]. 

HPAI viruses have also been isolated from free-flying wild birds; for example, 2 genotypes of HPAI (H5N1) viruses were isolated from apparently healthy tree sparrows (P. montanus) in China in 2003 and 2004 [5]. Also, large-scale outbreak of HPAI occurred in a zoological institution in Cambodia that affected a variety of captive wild-bird species including eagles and owls [7]. These findings suggested that land-based wild birds might also be a major reservoir of influenza viruses. Additionally, they intermingle freely with wild and domestic populations of waterfowl and poultry making them potentially important “bridge” in the transmission of avian influenza viruses from aquatic birds to poultry and vice versa. Therefore, these species may be relevant in virus transmission and should form an integral part of avian influenza surveillance and monitoring programs [8, 9]. But, there are limited data describing their vulnerability to influenza virus (H5N1) infection or potential to transmit the viruses [10]. 

In Nigeria, the highly pathogenic avian influenza (HPAI) virus of the H5N1 subtype was first detected in February 2006, in chickens at a commercial poultry farm in Kaduna state, Northern Nigeria. This was the first Africa's confirmed HPAI (H5N1) outbreak. The infection spread and persisted for 21 months, and a fatal human case was reported [11]. The AI viruses of the H5N2 subtypes had been detected by means of molecular tests in free-living and apparently healthy White-faced Whistling-duck (Dendrocygna viduata) and Spur-winged Goose (Plectropterus gambensis) [12] in Nigeria; supporting the view that migratory birds may serve an important role in the introduction of HPAI (H5N1) viruses into Nigeria. Introduction and spread of the virus through illegal trade in poultry and poultry products have not been ruled out [13]. Illegal poaching of wild birds and other wild-life species for food, traditional medicine, and certain belief are not uncommon in Nigeria. Captive wild birds are usually kept in rural community setting for a while, awaiting market days and may in the process come in contact with domestic poultry. Karesh et al. [14] mention that trade in wildlife commonly provides disease-transmission avenues at scales that not only cause human disease outbreaks but also threaten livestock, international trade, rural livelihoods, native wildlife populations, and the health of ecosystems. 

From October 2007 to June 2008, despite intensive surveillance for AI, there had been no reported cases in Nigeria. This development helped to ease out fear among poultry farmers and had a positive effect on restoration of consumers' confidence in poultry products [15], but the relief was for a short period. In July 2008, new HPAI-virus isolates were obtained. Two of the isolates from Kano and Katsina states belonged to clade 2.2, previously isolated in Nigeria, but isolates from Gombe and Kebbi states were of a new sublineage of clade 2.2.1, which was new to the African continent [13, 15]. 

The factors responsible for the spread and sustenance of the HPAI viruses in infected states of Nigeria are still not clear. The issue is, do avian influenza viruses persist and circulate at reasonably detectable levels in apparently healthy potential “bridge” species of birds, in Nigeria? Ultimately, the objective of this study was to determine whether avian influenza viruses were present at reasonably detectable levels (prevalence of 0.5%) in apparently healthy possible “bridge” species of wild and domestic birds around poultry houses and in captive wild birds markets. It was envisaged that the study would bring about better understanding of the possible roles played by “bridge” species in the re-occurrence and spread of avian influenza viruses among poultry. As well as early detection of the possible risks posed by their activities to the poultry sector. Findings would also be useful in setting priorities for surveillance and evaluation of control programmes.

## 2. Materials and Methods

### 2.1. Target Species

The target was wild birds, scavenging around active poultry houses, moving inbetween poultry farms, and that might come intact with aquatic birds at water bodies and rice fields. Also, species in captivity are sold in live wild birds markets. Majority of wild birds in captivity were captured at the wetlands or acquired from other sources such as neighbouring countries. These categories were considered as potential “bridge” between aquatic birds and domestic poultry.

### 2.2. Survey Design

Using available and relevant information such as lists of states, infected poultry farms, and wild bird markets; a multistage simple random-sampling technique with cluster sampling at primary sampling units was adopted. Briefly, 8 states (Borno, Gombe, Kaduna, Kano, Plateau, Ogun, Oyo, and Yobe) were selected at random from the list of 25 avian influenza infected states. In each selected state, 1 poultry farm was selected from list of previously infected but restocked farms, then, all wild birds trapped by mist net around the farm were sampled. Also, in selected sates that had captive live wild birds markets, 1 captive wild bird trader was selected randomly from the list of traders in each market, and all wild birds in his position were sampled. In total, 8 poultry farms and 8 wild birds' markets were surveyed.

As data on population sizes are lacking in wild animals, including wild birds species, due to their remoteness, varying populations, and the migratory behaviour these animals constitute a major challenge for designing disease monitoring systems [16]. Furthermore, in this study, prevalence of avian influenza viruses in the targeted species in Nigeria was unavailable and it was considered unreasonable to extrapolate based on prevalence obtained in other places. Therefore, as in majority of similar studies conducted elsewhere [6, 9, 12, 17–19], it was considered convincible to constrain the sample size to available resources. 

The team for the study was composed of veterinarians, disease surveillance agents, laboratory diagnosticians, and an ornithologist. In collaboration with FAO Nigeria and Rome and the Ornithological Research Institute, Jos, the team was given a refreshing training on biosecurity measures, sensitization of poultry farmers, captive wild-bird traders, leaders of local communities, and on capturing and handling wild birds, sampling, and sample preservation. All activities were simulated by participants on the last day of the training and appropriate corrections made on observed lapses. The study was carried out between August and September 2008. Prior to the commencement of field activities, owners of all selected poultry farms, leaders of traders, and the host communities were contacted, visited, and sensitized on the objectives of the study, its benefits, and their expected roles such as winning the cooperation of their workers and members. Also, the schedules for the survey exercise was discussed with them and agreed upon.

**Figure 1:**
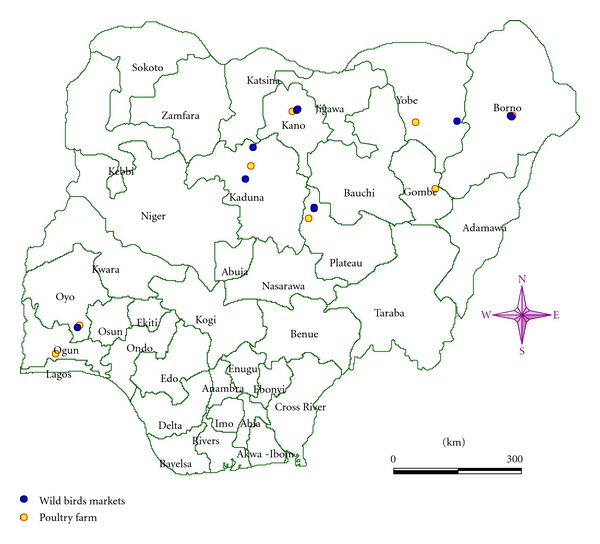
Distribution of sampling sites for poultry farms and wild birds markets.

Visits to poultry farms were made twice every day, early in the morning and late in the evening from about 5:30 to 8:30 AM and from 5:10 to 7:10 PM (local time), respectively, being periods of maximum activities for majority of birds and less stress to birds during handling. Birds were captured using mist nets, harvested and sampled according to standard practices [18, 20]. Both pharyngeal and cloacal swabs collected from each bird were stored separately in a cryovial tube containing viral transport media as described by Gaidet et al. [21]. Briefly, the viral transport media consisted of isotonic phosphate-buffered saline, pH 7.0–7.4, containing antimicrobial agents (penicillin 10,000 U/mL, streptomycin 10 mg/mL, amphotericinB 25 *μ*g/mL, and gentamycin 250 *μ*g/mL) supplemented with 10% glycerol. Samples were labelled (date, species of birds, sample type (tracheal or cloacal), and specific identity number) using indelible marker. Aseptically procedures were maintained using 70% alcohol. Same procedures were followed in captive wild birds markets. The coordinates of the sample points were recorded using Garmin eTrex legend by Garmin International Inc., USA. See map in Figure 1. Samples were parked, stored on ice park in a colman box, sealed, and transported to the laboratory with completed data forms at most within 36 hours of collection. Samples were stored at −80°C in the laboratory pending testing. The cold chain was maintained throughout the processes.

All samples were tested at the National Veterinary Research Institute (NVRI), Vom, using viral isolation in embryonated chicken eggs in line with standard procedures [22] and described by Joannis et al. [15]. Virus isolation was done in 9–11-day-old embryonated chicken eggs (incubated at 37°C for 5 days). The eggs were candled daily to determine viability and eggs with dead embryos were removed and kept at +4°C. All eggs were opened aseptically and the allantoic fluids (ALFs) were harvested and tested by haemagglutination test. The chorioallantoic membranes (CAMs) of positive eggs were tested by agar gel immunodiffusion (AGID) to detect influenza A virus group antigen and haemagglutination inhibition (HI) test to determine the virus subtype. All negative ALF (from all the samples in this case) were further passaged in a second set of embryonated chicken eggs. Any negative samples after the second passage were declared as such. The calculations for the confidence interval were done in R 2.10.1 program for exact binomial CI () function. Other calculations were done using Microsoft Excel 2003.

## 3. Results

A total of 403 birds made up of 40 species were sampled. 6 samples (afterthought) were positive for newcastle disease virus (NDV) but all tested samples were negative for avian influenza viruses. Therefore, the confidence interval (CI) was calculated at 95% limit. The overall CI was 0–0.007406 (upper and lower limits, resp.). Among the states, Ogun had the lowest samples 6.9%, CI (0–0.101466); Plateau the highest 21.6%, CI (0–0.033848). In the overall species, sampled, Tawny Eagle (Aquila rapax) was the lowest sampled 0.3% and Red-billed Firefinch (Lagonosticta senegala) was the highest 11.7%. Among the 281 wild birds category sampled, Tawny Eagle (A. rapax) was the lowest sampled 0.4%, CI (0–0.95); while the highest was Red-billed Firefinch (L. senegala) 16.7%, CI (0–0.06175). In domestic birds category, 122 were sampled, out of this, Black-crowned Crane (Blearica pavonina) (in captivity) was the lowest sampled 1.6%, CI (0–0.776393); Domestic Duck (Anas platyrhynchos) was the highest sampled 35.3%, CI (0–0.067297). In both cases, the lowest sampled had wider CI, an indication of higher uncertainty, while the highest sampled had relatively narrower CI indicative of relatively lesser uncertainty. This may be attributable to differences in the sample sizes, as bigger sample size tends to reduce the uncertainty in a survey. Determination of confidence limits for animal disease monitoring results has important applications. It can be applied in the verification or rejection of a hypothesis about a given system and in describing the proportion of the disease impact that remains undetected and which may lead or contribute to unobserved spread of a disease [16], in this study, the latter may be considered applicable. The details of the results are as depicted on Table 1.

## 4. Discussion 

The manifestation, host invasiveness, ecological spread, and associated risks of highly pathogenic avian influenza (HPAI) H5N1 subtype have resulted in increased concerns globally. Similarly, the devastating agricultural and economic problems encountered in domestic poultry subsector, as well as fatal human cases resulting from the infection, have been substantially overwhelming [5]. The situation is worse in majority of developing countries, where poultry rearing particularly at rural areas is a major source of livelihood and in majority of cases the most viable and sustainable source. Over the years, the complex role played by wild birds in the introduction, spread, sustenance, and incidences of avian influenza viruses particularly H5N1 has been a subject for in-depth exploration.

Surveillance for avian influenza viruses in wild and domestic birds provides an exclusive opening to enhance our knowledge not only of ecology and epidemiology of the HPAI virus subtypes but also that of LPAI viruses in their natural hosts, at the same time and for the same cost. This approach enables timely detection of palpable signals for the introduction of the disease into new regions and may provide access to updated data and strains of the viruses for categorization. However, for proper evaluation of control programmes, risk-based decision making, and prioritization of utilization of scarce resources, we also need a better understanding of the roles of interfaces between wild and domestic birds. For example, we need clearer insight of the possible role played by potential “bridge” species of wild and domestic birds, aimed at the identification of virus permissiveness of these species and their relative likelihood to develop disease and patterns of virus secretion [9].

The occurrence and distribution of subtypes of influenza A viruses broadly, may vary between different surveillance studies depending on target species, time, and place [6, 23]. Previous studies have attributed this phenomenon to differences in taxonomy and behaviour such as colony breeding, feeding patterns, and aggregation at stopover or wintering sites which encourages mixing of different population of birds. Also the deferential attention by researchers to aquatic birds' species believed to harbour the viruses. For example, previous studies have demonstrated that virus isolations from other wild birds have been completely surpassed by the number, variety, and widespread distribution of influenza viruses in waterfowl, order Anseriformes [24, 25]. This could be a possible explanation for limited data on avian influenza viruses in other wild birds species particularly land-based species.

It is acknowledged that this study had short-comings of relatively small sample size, attributable to limited resources particularly funds available for the study, number of days allotted for the study, and the timing. While the limitation of resources on the study was obvious, that of timing was also prominent. The study was circumstantially conducted during the raining season. A period when possible “bridge” species targeted had alternative sources of water and feeds. As such, were dispersed and made less frequent visits in search for feeds to poultry houses where majority of captures were made. More so, the timing coincided with period of increased demand for poultry eggs in Nigeria. Therefore, farmers would not allow extended surveillance activities around their farms for fear that the birds would be disturbed which might lead to drop in production. This contributed to the limitation of the sample size as well. Also, the limitations of designing effects including clustering had not been accounted for due to the all negative outcome (lack of variance) of the data. This result could as well have been influenced by factors such as high virulence of HPAI virus in these species, such that infected birds could not survive long enough, extremely low prevalence of the viruses in these species that lead to failed detection, or the outcome was by chance. For instance, unlike aquatic wild birds that congregate during breeding seasons and at roosting sites leading to the build up of a local population density that facilitates spread of diseases through contact, land-based wild birds rarely congregate at particular spots but are dispersed majority of the time, allowing less time for spread of diseases through contact. It could as well be that these species lacked the ability to harbour the viruses particularly LPAI subtypes. May be lack of carrier status for the viruses? This could serve as a subject of considerable interest for further study. However, the 95% confidence intervals for the data so obtained were calculated. This was with the assumption that if the study had been repeated several times, the true values of the data would have been captured within the confidence intervals.

The outcome of the study reflected the varied outcomes of similar studies conducted elsewhere. For example out of more than 10,000 samples collected from wild birds in 15 orders other than the Anseriformes, Charadriiformes, and Gruiformes, no influenza A viruses were detected [26]. In contrast, in another study out of 939 samples from land-based wild birds, order Passeriformes, 24 were positive for influenza A viruses giving a prevalence of 2.6% [9]. The latter, establishes the possible occurrence of these viruses in wider wild birds species other than aquatic birds and suggests the need for widening up of surveillance activities to incorporate more species. It seems most likely (at least from previous studies) that the situation of avian influenza viruses in land-based wild birds which form majority of possible “bridge” species remain poorly understood or largely unknown and need to be explored. The need for this is more urgent in developing countries where contacts between wild birds and domestic poultry are more rampant than would be expected due to the production system. 

To obtain a clearer picture of avian influenza viruses in potential “bridge” species of birds, a multidisciplinary approach with effective collaborations is required. This has the advantage of maximising the utilization of scarce human expertise and financial resources particularly in developing countries. For example a team could be composed of a veterinarian, statistician, ornithologist, laboratory diagnostician, and rural sociologist. This approach, combined with a community-oriented approach to disease reporting, would most probably yield the desired result of better understanding. While the human communal life style in African villages encourages the comingling of household flocks, it, most importantly, provides the social setting convenient for collective action to prevent diseases, improve poultry production systems [27], and disease reporting.

The major contributions of this study are as follows: firstly, targeted surveillance of avian influenza viruses in this group of birds should be given special consideration of supplementation with extensive passive surveillance normally targeting sick or recently dead birds. This view is consistent with the findings by Hesterberg et al. [17] and Feare [28] that active surveillance for avian influenza viruses is essential in providing important information on LPAI viruses' circulation, including H5 and H7 subtypes that have the potential to become highly pathogenic in poultry. While vigilance for dead birds through passive surveillance may remain the best means of detecting HPAI viruses such as H5N1 subtypes in wild birds. The possible explanation to this theory which also concurs with that highlighted by Breed et al. [29] is that; on one hand, passive surveillance is more responsive for the detection of HPAI viruses, as birds may be either sick or dead. However, the limitation is that the method seems to exclude the detection of the HPAI viruses in healthy birds that may harbour them. On the other hand, active surveillance appears more responsive for the detection of LPAI viruses, as the viruses are confined, compared with the overwhelming HPAI viruses resulting in a lower chance of finding LPAI viruses in dead birds. However, Knight-Jones et al. [30] believe that sentinel surveillance is effective in detection of both LPAI and HPAI viruses. Integrating the two surveillance methods (passive and active), therefore, seems reasonable and may be more result oriented.

Secondly, the study identified 40 potential “bridge” species of birds for the spread of avian influenza viruses comprising those that frequented active poultry houses, and species that were sold in live wild birds markets. This may serve as base-line information for more elaborate study in Nigeria and elsewhere in future targeting these species. Majority of species trapped were seed predators (granivores), since poultry feed constitutes mainly of grains. However, because of the location of certain farms plus their integrated nature, some insectivorous birds like cattle egret and brown babblers were also trapped.

Thirdly, as mentioned in the result, the afterthought (not initially considered in the study) findings of 6 positive samples for Newcastle disease virus (NDV), apart from being an indication that samples were in good conditions, it suggested that two or more closely related diseases could be studied concurrently in wild birds. This approach particularly in developing countries has the advantage of being less time consuming, more cost effective, and informative. More so, isolation of NDV further implicated wild bird's species in the spread of other diseases like Newcastle disease. This scenario has contributed to complications in behavioural changes due to wrong perception of avian influenza, particularly amongst rural populations. They find it extremely difficult to believe that avian influenza is different from Newcastle disease. A disease they are used to and have lived with for long.

## 5. Conclusion

The study motivated by existing concerns on avian influenza has demonstrated surveillance for avian influenza viruses in wild and domestic birds' species that might serve as “bridge” at the interfaces between migratory birds and domestic poultry. The strength of the data might have been reduced by limitations including sampling size. Thus, it calls for caution in making strong conclusions and corroborates the need for broader coverage and blending of active and passive surveillances for avian influenza viruses in these species. Considering the dynamic nature of the avian influenza viruses, it would seem most probable that future study in this area will require concerted efforts, holistic and multidisciplinary approach focusing on appropriate ornithological and epidemiological variables such as type, census, and movement of birds, and distribution of avian influenza incidences in time and space in high-risk areas. These approaches, over a longer time frame, are likely to convey a better understanding of the possible roles played by potential “bridge” species of birds in the epidemiology of avian influenza viruses in Nigeria. This has implications for making informed decisions.

## Figures and Tables

**Table 1:** Category (states, type of bird (wild or domestic) species), number tested, and the confidence interval.

Category	Number tested	95% Confidence interval		Tested birds (%)*
Overall (All states)	403	0	0.007406	100
States				
Ogun	28	0	0.101466	6.9
Oyo	35	0	0.082032	8.7
Borno	43	0	0.067297	10.7
Yobe	27	0	0.105019	6.7
Kano	62	0	0.04717	15.4
Kaduna	75	0	0.039156	18.6
Plateau	87	0	0.033848	21.6
Gombe	46	0	0.063049	11.4

Type of birds (Wild species)				

Ethiopian Swallow (*Hirundo aethiopica*)	12	0	0.220922	4.3
White Stork (*Ciconia ciconia*)	2	0	0.776393	0.7
Tawny Eagle (*Aquila rapax*)	1	0	0.95	0.4
Chestnut-bellied Starling (*Lamprotornis pulcher*)	9	0	0.283129	3.2
Northern Red Bishop (*Euplectes orix*)	16	0	0.17075	5.8
Buffalo Weavers (*Bubalornis albirostris*)	9	0	0.283129	3.2
Pin-tailed Whydah (*Vidua macroura*)	2	0	0.776393	0.7
African Grey Parrot (*Psittacus erithacus*)	5	0	0.45072	1.8
Village Indigobird (*Vidua chalybeata*)	2	0	0.776393	0.7
Silverbill Bird (*Lonchura malabarica*)	4	0	0.527129	1.4
Senegal Parrot (*Poicephalus senegalus*)	11	0	0.238404	3.9
Rose-ringed Parakeet (*Psittacula krameri*)	14	0	0.192636	5
Four-banded Sandgrouse (*Pterocles quadricinctus*)	6	0	0.393038	2.1
Red-billed Firefinch (*Lagonosticta senegala*)	47	0	0.06175	16.7
Common Bulbul (*Pycnonotus barbatus*)	3	0	0.631597	1.1
Red-cheeked Cordon-bleu (*Uraeginthus bengalus*)	38	0	0.075808	13.5
Cattle Egret (*Bubulcus ibis*)	4	0	0.527129	1.4
Black-winged Red Bishop (*Euplectes hordeaceus*)	6	0	0.393038	2.1
Brown Babbler (*Turdoides plebejus*)	5	0	0.45072	1.8
Vinaceous Dove (*Streptopelia vinacea*)	7	0	0.348164	2.5
Yellow-fronted Canary (*Serinus mozambicus*)	8	0	0.312344	2.8
Cut-throat (*Amadina fasciata*)	2	0	0.776393	0.7
Bronze Mannikin (*lonchura cucullata*)	11	0	0.238404	3.9
Tawny-flanked Prenia (*Prinia subflava*)	5	0	0.45072	1.8
Laughing Dove (*Streptopelia senegalensis*)	5	0	0.45072	1.8
Rock Firefinch (*Lagonosticta sanguinodorsalis*)	2	0	0.776393	0.7
Village Weaver (*Ploceus cuculltus*)	33	0	0.086781	11.7
Yellow White-eye (*Zosterops senegalensis*)	2	0	0.776393	0.7
Winding Cisticola (*Cisticola galactotes*)	3	0	0.631597	1.1
Black-rumped Waxbill (*Estrilda troglodytes*)	3	0	0.631597	1.1
Grey-headed Sparrow (*Passer griseus*)	4	0	0.527129	1.4

Total	281	0	0.010604	100

Domestic and captive species				

Domestic Duck (*Anas platyrhynchos*)	43	0	0.067297	35.3
Helmeted Guineafowl (*Numida meleagris*)	26	0	0.10883	21.3
Black-crowned Crane (*Balearica pavonina*)	2	0	0.776393	1.6
Spur-winged Goose (*Plectropterus gambensis*)	11	0	0.238404	9
Canada Goose (*Branta canadensis*)	15	0	0.181036	12.3
Feral Pigeon (*Columba livia domestica*)	13	0	0.205817	10.7
Congo Peacock (*Afropavo congensis*)	2	0	0.776393	1.6
Little button Quail (*Turnix sylvatica*)	7	0	0.348164	5.7
Purple Swamphen (*Porphyrio porphyrio*)	3	0	0.631597	2.5

Total	122	0	0.024256	100

*Number of birds tested expressed as a percentage of the overall for states and percentage of totals for wild and domestic species.
